# A multicenter evaluation of the QIAstat-Dx meningitis-encephalitis syndromic test kit as compared to the conventional diagnostic microbiology workflow

**DOI:** 10.1007/s10096-024-04751-9

**Published:** 2024-01-11

**Authors:** Stefan A. Boers, Robin van Houdt, Nina M. van Sorge, Jelle Groot, Yvette van Aarle, Mario J. A. W. M. van Bussel, Louise F. E. Smit, Els Wessels, Eric C. J. Claas

**Affiliations:** 1 Dept. Medical Microbiology, Leiden University Medical Center, Albinusdreef 2, 2333 ZA Leiden, The Netherlands; 2 Dept. Medical Microbiology and Infection prevention, Amsterdam UMC location University of Amsterdam, Meibergdreef 9, 1105 AZ Amsterdam, The Netherlands; 3 Netherlands Reference Laboratory for Bacterial Meningitis, Amsterdam UMC location AMC, Meibergdreef 9, 1105 AZ Amsterdam, The Netherlands

**Keywords:** Syndromic testing, QIAstat-Dx, Central nervous system (CNS) infections, Meningitis, Encephalitis

## Abstract

**Purpose:**

Rapid diagnosis and treatment of infectious meningitis and encephalitis (ME) is critical to minimize morbidity and mortality. Recently, Qiagen introduced the CE-IVD QIAstat-Dx ME panel (QS-ME) for syndromic diagnostic testing of meningitis and encephalitis. Some data on the performance of the QS-ME in comparison to the BioFire FilmArray ME panel are available. In this study, the performance of the QS-ME is compared to the current diagnostic workflow in two academic medical centers in the Netherlands.

**Methods:**

A total of 110 cerebrospinal fluid samples were retrospectively tested with the QS-ME. The results obtained were compared to the results of laboratory-developed real-time PCR assays (LDTs), IS-pro, bacterial culture, and cryptococcal antigen (CrAg) testing. In addition, the accuracy of the QS-ME was also investigated using an external quality assessment (EQA) panel consisting of ten samples.

**Results:**

Four of the 110 samples tested failed to produce a valid QS-ME result. In the remaining 106 samples, the QS-ME detected 53/53 viral targets, 38/40 bacterial targets, and 7/13 *Cryptococcus neoformans* targets. The discrepant bacterial results consisted of two samples that were previously tested positive for *Listeria monocytogenes* (C_T_ 35.8) and *Streptococcus pneumoniae* (C_T_ 40), respectively. The QS-ME detected one additional result, consisting of a varicella-zoster virus signal (C_T_ 35.9), in a sample in which both techniques detected *Streptococcus pyogenes*. Finally, 100% concordance was achieved in testing a blinded bacterial ME EQA panel.

**Conclusion:**

The QS-ME is a relevant addition to the syndromic testing landscape to assist in diagnosing infectious ME.

**Supplementary information:**

The online version contains supplementary material available at 10.1007/s10096-024-04751-9.

## Introduction

Rapid diagnosis of infectious diseases results in optimized treatment and improved patient outcomes. Syndromic testing panels, covering a wide range of causative pathogens, have been available for several years for respiratory infections, gastroenteritis, and positive blood cultures. An important infectious syndrome is meningitis and encephalitis (ME), as this syndrome is associated with serious sequelae and mortality. The etiological agents can be viruses, bacteria, and fungi, requiring a diverse set of tests including bacterial culture, real-time PCR, and antigen tests, and as a consequence, a final result may take days [1]. In 2016, the first reports had been published on the diagnostic application of the BioFire® FilmArray® ME panel (FA-ME) (BioMérieux, Lyon, France), which simultaneously detects 14 different pathogens involved in central nervous system (CNS) infections [2, 3]. Recently, two systematic reviews have been published on the application of the FA-ME, and in general, the shortened time to a diagnostic result improved antimicrobial and antiviral treatment, and resulted in earlier hospital discharge leading to a reduction in costs per patient [4, 5].

In 2022, a second syndromic panel for ME has been launched, the QIAstat-Dx® ME panel (QS-ME) (Qiagen, Hilden, Germany). The QS-ME test is a cartridge-based multiplex real-time PCR test that detects the bacterial pathogens *Escherichia coli* K1, *Haemophilus influenzae*, *Listeria monocytogenes*, *Neisseria meningitidis*, *Streptococcus agalactiae* (Group B *Streptococcus*), *S. pneumoniae*, *S. pyogenes* (Group A *Streptococcus*), and *Mycoplasma pneumoniae*. In addition, the viral pathogens enterovirus, herpes simplex type 1 (HSV-1) and type 2 (HSV-2), human herpesvirus 6 (HHV-6), parechovirus, and varicella zoster virus (VZV) are included, and the yeasts *Cryptococcus neoformans* and *Cryptococcus gattii* are both detected, but not differentiated*.* The main difference between the assays from BioMérieux and Qiagen is that the QS-ME lacks cytomegalovirus (CMV) but contains *S. pyogenes* and *M. pneumoniae*. Furthermore, the QS-ME provides PCR curves and C_T_ values for qualitative and semi-quantitative evaluation of results.

Recently, three publications compared the two syndromic tests and all three studies showed highly concordant results with combined positive percent agreements of > 95% and negative percent agreements of > 94% between both tests [6–8].

In the current study, the QS-ME was applied to a retrospective set of 110 cerebrospinal fluid (CSF) samples that had previously been tested positive for one of the QS-ME targets by the current laboratory workflows in two academic medical centers in the Netherlands, one of which is the Netherlands Reference Laboratory for Bacterial Meningitis as well. Only samples with sufficient remnant CSF for testing the QS-ME panel as well as re-testing our current, real-time PCR-based workflow were selected. To get a more detailed insight in the QS-ME performance, a specificity panel comprising four CSF samples and an external quality assessment (EQA) panel were also included in the assay analysis.

## Materials and methods

### Clinical samples

The study has been performed using remnant CSF samples obtained by lumbar puncture submitted to the clinical microbiology laboratories for diagnosing patients suspected of a neurological infection. Overviews of CSF-positive samples were generated using the laboratory information systems. The selection was performed to cover all pathogens detected by the QS-ME. Only the *M. pneumoniae* QS-ME target could not be evaluated in this study as no *M. pneumoniae*-positive samples were available. All samples had been previously tested by real-time PCR, IS-pro, culture, or cryptococcal antigen (CrAg) testing in our ISO15189 accredited laboratories as described below. For the viral targets, only samples with sufficient volume left to perform both the QS-ME and retest by in-house real-time PCR have been included. The latter was performed to exclude variation in sample integrity as the samples had been collected in 2018 and 2019 and stored at − 80 °C. Only samples from patients that indicated no objection for further use of their samples were included. The samples were anonymized and no clinical data were used, omitting the need for approval by an ethical committee.

### QIAstat-Dx® meningitis-encephalitis test

The assay was performed according to the instructions for use (IFU). In short, 200 µl CSF was transferred to the main port of the cartridge using the included transfer pipettes. After scanning the barcode of the sample and the cartridge in the QIAstat-Dx Analyzer 1.0, the cartridge was transferred to the analyzer. After starting the test, extraction, amplification, and detection of the nucleic acids (NA) were automatically performed by the QIAstat-Dx Analyzer 1.0. The QS-ME cartridge includes an internal control (IC) that verifies all steps of the procedure. The lack of an IC signal implied that negative results were invalid. Results were available 80 min after starting the assay.

### Bacterial culture and CrAg testing

CSF was inoculated at 35 °C for 5 days on trypticase soy agar (TSA) plates with 5% sheep blood (BioMérieux) and chocolate agar PolyViteX plates (BioMérieux) under 5% CO_2_ conditions, TSA plates with 5% sheep blood (BioMérieux) under anaerobic conditions, in thioglycolate with resazurin broth (BioMérieux), and in tryptone soy broth with XV factor (Mediaproducts). Cryptococcal cultures were incubated on sabouraud supplemented with chloramphenicol and gentamicin agar (BioMérieux) at 28 °C and 35 °C for 14 days and a subculture of the inoculated tryptone soy broth incubated at 35 °C. CrAg testing was performed using Murex Cryptococcus latex agglutination assay (Remel, Lenexa, USA) and Dynamiker Cryptococcal Antigen Lateral Flow Assay (Dynamiker Biotechnology, Tianjin, China). No PCR-based assay had been implemented for *Cryptococcus* spp. These samples were selected based on positive culture results (*n* = 5) or positive CrAg tests (*n* = 9).

### PCR-based assays

The current diagnostic workflow is based on laboratory-developed real-time PCR assays (LDTs) that have been implemented in the laboratories under ISO15189:2012 accreditation for detection of bacterial [9–12] and viral pathogens [13–17]. IS-pro was used for uncommon bacterial pathogens. IS-pro is a CE-marked bacterial profiling technique, focusing on species-specific variations in the length of PCR fragments of the 16S-23S interspace region [18].

For the study, NA were extracted from 200 µl CSF using MagNA Pure 96 DNA and Viral NA small volume kit on the MagNA Pure 96 extraction platform (Roche Diagnostics, Almere, The Netherlands). Subsequently, 10 µl NA was subjected to the respective reagent mix and protocol for the detection of the requested pathogens. Amplification was performed either on BioRad CFX96 real-time PCR platforms (BioRad, Veenendaal, The Netherlands) or on the LightCycler 480 Instrument II (Roche Diagnostics, Almere, The Netherlands).

### Enterovirus genotyping

Genotyping of enteroviruses was performed by sequencing the VP1 capsid protein region [19]. PCR amplicons were submitted to Macrogen (Amsterdam, The Netherlands) for Sanger sequencing. The generated ABI format chromatogram files were used to generate a FASTA file for analysis using the enterovirus genotyping tool v 1.0 (RIVM, Bilthoven, The Netherlands) [20].

## Results

Initially, a total of 110 CSF samples were tested by the QS-ME and retested by LDTs. Four samples resulted in an invalid QS-ME result, either due to a negative internal control signal (one sample) or cartridge failures (three samples). These samples could not be re-tested due to insufficient sample volume and were excluded from further analysis. Fifty-three of the remaining 106 CSF samples tested positive for viral targets with LDTs, all of which were also detected by the QS-ME (Table 1). In addition, a total of 40 CSF samples that previously tested positive for bacterial targets and 13 CSF samples that tested positive for the fungus *C. neoformans* were used to test the QS-ME performance. There was concordance for 38 out of 40 (95%) of these bacterial targets. There was one bloody CSF sample that tested positive (C_T_ value 35.8) for *L. monocytogenes* in the LDT but was negative in the QS-ME. Despite the bloody appearance, the IC of the QS-ME was detected and so the result was valid. In the other discrepant sample, the QS-ME failed to detect an *S. pneumoniae* that was detected in the LDT with a C_T_ value of 40. Finally, only seven of the 13 samples positive for *C. neoformans* tested positive in the QS-ME.

**Table 1:** Comparison of viral, bacterial, and fungal targets detected in QS-ME and the concordance with the LDT, IS-pro, culture, and/or Cryptococcus Ag test results

QS-ME target	LDT, IS-pro, culture, and/or Cryptococcus Ag positive	QS-ME positive	Concordance
Enterovirus	24	24	100%
HHV-6	3	3	100%
HSV-1	6	6	100%
HSV-2	4	4	100%
Parechovirus	5	5	100%
VZV	11	12	92%
*E. coli* K1	1	1	100%
*H. influenzae*	7	7	100%
*L. monocytogenes*	7	6	86%
*M. pneumoniae*^a^	-	-	-
*N. meningitidis*	10	10	100%
*S. agalactiae*	1	1	100%
*S. pneumoniae*	11	10	91%
*S. pyogenes*	3	3	100%
*C. neoformans*	13	7	54%^b^

^a^The QS-ME *M. pneumoniae* target could not be evaluated in this study as there were no *M. pneumoniae* CSF-positive samples available

^b^The concordance between *Cryptococcus* culture and QS-ME was 50% (2/4) and for CrAg and QS-ME 56% (5/9)

Interestingly, one of the *S. pyogenes*-positive samples showed the detection of a second infectious agent in the QS-ME. Besides the confirmation of the *S. pyogenes* result (C_T_ 20.9), the QS-ME also detected a VZV signal with C_T_ 35.9. Unfortunately, no additional sample was available for retesting with the LDT.

An additional four CFS samples were tested to determine QS-ME test specificity. These include a negative CSF sample, two CSF-positive samples for a species from the *S. bovis* group, and one CSF-positive sample for *S. dysgalactiae*. All four CSF samples tested negative in the QS-ME (Table S1).

Although both the QS-ME and the LDT provide C_T_ values, the results cannot be directly compared without standardization. However, for all DNA targets, very comparable C_T_ values were observed with a median C_T_ value difference of 1.2, with no structural advantage for one of the workflows (Table S1). For the RNA targets, enterovirus and parechovirus, it was observed that in all but one sample, the QS-ME showed higher C_T_ values as compared to the LDTs. Although in most samples the difference was small (less than three C_T_ values), in eight out of 24 (33%) enterovirus-positive samples and two out of five (40%) parechovirus-positive samples, the QS-ME generated significantly higher C_T_ values (> 3 C_T_) as compared to the LDTs as shown in gray in Table S2 (supplementary material). Given the large amount of genotype variants in these virus families, it is possible that this contributed to less efficient detection of some viral subtypes. This hypothesis could however not be confirmed by additional genotyping performed on enterovirus-positive samples, as six out of eight (75%) of the enterovirus-positive samples with large discrepancies in C_T_ value were all found to contain echovirus E30, a genotype that was also found in samples with a comparable range in C_T_ value (Table S1). For parechoviruses, we do not have a genotyping method operational.

The accuracy of the QS-ME was also investigated using the 2022 CNS II (non-viral meningitis and encephalitis) EQA panel consisting of ten samples that were blinded and distributed by the Quality Control for Molecular Diagnostics (QCMD). As shown in Table 2, all EQA samples were correctly detected using the QS-ME.

**Table 2:** Comparison of test results with the QCMD EQA panel

Sample code	Sample content	QS result
CNSII22S-01	*S. pneumoniae*	*S. pneumoniae* (C_T_ 30.7)
CNSII22S-02	*N. meningitidis*	*N. meningitidis* (C_T_ 27.7)
CNSII22S-03	*E. coli* K1	*E. coli* K1 (C_T_ 29.6)
CNSII22S-04	*L. monocytogenes*	*L. monocytogenes* (C_T_ 33.2)
CNSII22S-05	*H. influenzae*	*H. influenzae* (C_T_ 25.1)
CNSII22S-06	*C. neoformans*	*C. neoformans/gattii* (C_T_ 33.2)
CNSII22S-07	*S. pneumoniae*	*S. pneumoniae* (C_T_ 31.6)
CNSII22S-08	*S. agalactiae*	*S. agalactiae* (C_T_ 34.7)
CNSII22S-09	Negative	Negative
CNSII22S-10	*H. influenzae*	*H. influenzae* (C_T_ 24.7)

## Discussion

Infections of the central nervous system can result in life-threatening conditions and severe longlasting debilitating sequelae. As the etiological microbiological agents are diverse, a proper diagnosis resulting in an appropriate treatment plan may take days. Given the impact of neurological infections, rapid, random access diagnostics would result in faster initiation of appropriate patient treatment [5]. The introduction of syndromic testing panels in molecular diagnostics, i.e., for respiratory infections, has been shown to improve patient management [21, 22]. Although for gastroenteritis the added value is less obvious [23], some studies do report better patient management using syndromic testing for gastroenteritis as well [24, 25].

Where the FA-ME has been around for many years [2], the QS-ME has been launched only recently. The workflow of the two assays is comparable. The run-time of the FA-ME assay is somewhat faster than the QS-ME (60 min for FA-ME, versus 76 min for QS-ME). On the other hand, the QS-ME generates PCR curves with C_T_ values. The first comparative studies showed similar results between the two assays [6–8], although the reported number of invalid analyses was higher using the QS-ME with a total error rate of 6% (7/115 tests) versus no FA-ME invalid analyses (0/155 tests) in one of the studies [6]. In our analysis of 124 samples (including 114 clinical samples and 10 EQA samples), four invalid results were observed (3% error rate). One of these invalid results was an internal control failure and three invalid results were caused by cartridge failures.

The QS-ME showed on average a concordance of > 97% for the viral and bacterial targets detected within CSF samples as compared to our current diagnostic workflow. However, CSF samples containing echovirus E30 showed heterogenous results with some samples giving similar results while others resulted in significantly higher C_T_ values using the QS-ME. As these strains belonged to the same genotype (i.e., echovirus E30), the differences in C_T_ values are unlikely explained by sequence heterogeneity in the primer/probe region. Another possibility is that the use of retrospective samples, that had been stored at − 80 °C, may have affected the integrity of the virus RNA in a manner that disproportionally affected QS-ME detection compared to LDT detection. Also, the use of different (reverse transcriptase) PCR mastermixes in the QS-ME compared to the LDT may have contributed to the difference in C_T_ values [26]. This needs to be studied in a prospective application of the QS-ME. Although no false positive results were found for various streptococci and sensitivity was in line with our LDT results, proper analysis of the specificity of the assay needs to be determined in a prospective study as well, where a substantial number of samples are expected to remain negative.

The good analytical results were confirmed by a 100% concordance in testing a blinded bacterial ME EQA panel. In contrast, the analytical performance of the *Cryptococcus* target reached a concordance of just 50% when comparing results to cryptococcal culture (*n* = 4) and 56% when comparing results to CrAg testing (*n* = 9), raising concerns about analytical sensitivity [27].

The added clinical value of positive QS-ME results for patient management is evident. However, the interpretation of negative results might prove to be more challenging, especially for bacteria, as a negative result will unlikely result in the withdrawal of antibiotics. For example, the QS-ME (and the FA-ME) detects *E. coli* strains possessing the K1 capsular polysaccharide only. Although *E. coli* K1 isolates are predominant in (neonatal) *E. coli* meningitis, an estimated 20% do not carry the specific target for the QS-ME, resulting in potential false reassurance in a negative QS-ME result [28]. On the other hand, QS-ME negative results for viral targets probably mean that antiviral treatment will not be started or ceased.

This study has several limitations related to the relatively small number of CSF samples that could be included due to limited availability. For one QS-ME target (i.e., *M. pneumoniae*), there was no positive sample available, and some QS-ME targets had only a few representatives that were too low for a proper assessment of the QS-ME. In addition, only a few negative samples were selected to assess the QS-ME specificity. In future prospective studies, the number of negative samples encountered in daily routine would be high, from which a reliable percent negative agreement could then be calculated.

In conclusion, the QS-ME offers a comprehensive panel for pathogen detection in CSF samples using a simple sample-to-answer format with minimal hands-on time and a relatively short turnaround time. Therefore, the QS-ME represents a new and good alternative to the use of LDTs, similar to FA-ME, for detecting the most common viral and bacterial causative pathogens of ME. However, based on our data, QS-ME would not be recommended as a standalone diagnostic test for cryptococcal meningitis. Although implementation of the QS-ME will improve patient management by faster initiation of evidence-based patient management, the cost and clinical efficacy need to be determined in proper cost-benefit and qualitative evaluation studies.

### Supplementary information

Below is the link to the electronic supplementary material.

Supplementary file1 (DOCX 41 KB)

## Data Availability

Not applicable.
