# Supplementary material for: A multicenter evaluation of the QIAstat-Dx meningitis-encephalitis syndromic test kit as compared to the conventional diagnostic microbiology workflow
Source: Eur J Clin Microbiol Infect Dis. 2024 Jan 11;43(3):511–6. doi: 10.1007/s10096-024-04751-9 (PMC10917839; doi:10.1007/s10096-024-04751-9)
Supplement: Supplementary file 1 — Supplementary file1 (DOCX 41 KB) [file 10096_2024_4751_MOESM1_ESM.docx]

**Supplementary Information**

**Table S1: Overview of results obtained with QS-ME and routine diagnostic workflows.**

| **Institute** | **Sample ID** | **Routine diagnostic workflow** | | | **QS-ME** | | **Agreement** |
| --- | --- | --- | --- | --- | --- | --- | --- |
|  |  | **Assay** | **Result** | **C_T_** | **Result** | **C_T_** |  |
| AUMC | 1 | Ag test | *C. neoformans* | NA | Negative | NA | **NO** |
| AUMC | 2 | Ag test | *C. neoformans* | NA | *C. neoformans/gattii* | 33.2 | **YES** |
| AUMC | 3 | Ag test | *C. neoformans* | NA | *C. neoformans/gattii* | 23.3 | **YES** |
| AUMC | 4 | Culture | *C. neoformans* | NA | *C. neoformans/gattii* | 24.5 | **YES** |
| AUMC | 5 | Culture | *C. neoformans* | NA | Negative | NA | **NO** |
| AUMC | 6 | Culture | *C. neoformans* | NA | Internal control failure | NA | **EXCLUDED** |
| AUMC | 7 | Culture | *C. neoformans* | NA | *C. neoformans/gattii* | 27.4 | **YES** |
| AUMC | 8 | Culture | *C. neoformans* | NA | Negative | NA | **NO** |
| LUMC | 9 | Ag test | *C. neoformans* | NA | Negative | NA | **NO** |
| LUMC | 10 | Ag test | *C. neoformans* | NA | *C. neoformans/gattii* | 31.4 | **YES** |
| LUMC | 11 | Ag test | *C. neoformans* | NA | Negative | NA | **NO** |
| LUMC | 12 | Ag test | *C. neoformans* | NA | *C. neoformans/gattii* | 34.0 | **YES** |
| LUMC | 13 | Ag test | *C. neoformans* | NA | Negative | NA | **NO** |
| LUMC | 14 | Ag test | *C. neoformans* | NA | *C. neoformans/gattii* | 34.5 | **YES** |
| AUMC | 15 | Culture | *E. coli* | NA | Cartridge failure | NA | **EXCLUDED** |
| AUMC | 16 | Culture | *E. coli* | NA | *E. coli* K1 | 27.7 | **YES** |
| LUMC | 17 | LDT PCR | Enterovirus | 29.9 | Enterovirus | 31.9 | **YES** |
| LUMC | 18 | LDT PCR | Enterovirus | 31.0 | Enterovirus | 34.3 | **YES** |
| LUMC | 19 | LDT PCR | Enterovirus | 31.7 | Enterovirus | 33.4 | **YES** |
| LUMC | 20 | LDT PCR | Enterovirus | 28.4 | Enterovirus | 37.2 | **YES** |
| LUMC | 21 | LDT PCR | Enterovirus | 29.2 | Enterovirus | 39.4 | **YES** |
| LUMC | 22 | LDT PCR | Enterovirus | 31.6 | Enterovirus | 33.6 | **YES** |
| LUMC | 23 | LDT PCR | Enterovirus | 27.3 | Enterovirus | 36.4 | **YES** |
| LUMC | 24 | LDT PCR | Enterovirus | 33.9 | Enterovirus | 35.8 | **YES** |
| LUMC | 25 | LDT PCR | Enterovirus | 32.6 | Enterovirus | 34.9 | **YES** |
| LUMC | 26 | LDT PCR | Enterovirus | 34.8 | Enterovirus | 38.2 | **YES** |
| LUMC | 27 | LDT PCR | Enterovirus | 32.4 | Enterovirus | 33.7 | **YES** |
| LUMC | 28 | LDT PCR | Enterovirus | 29.8 | Enterovirus | 37.9 | **YES** |
| LUMC | 29 | LDT PCR | Enterovirus | 33.0 | Enterovirus | 34.9 | **YES** |
| LUMC | 30 | LDT PCR | Enterovirus | 32.2 | Enterovirus | 34.2 | **YES** |
| LUMC | 31 | LDT PCR | Enterovirus | 35.6 | Enterovirus | 37.0 | **YES** |
| LUMC | 32 | LDT PCR | Enterovirus | 32.4 | Enterovirus | 35.8 | **YES** |
| LUMC | 33 | LDT PCR | Enterovirus | 23.2 | Enterovirus | 23.3 | **YES** |
| LUMC | 34 | LDT PCR | Enterovirus | 25.6 | Enterovirus | 26.7 | **YES** |
| LUMC | 35 | LDT PCR | Enterovirus | 24.7 | Enterovirus | 26.6 | **YES** |
| LUMC | 36 | LDT PCR | Enterovirus | 30.5 | Enterovirus | 31.5 | **YES** |
| LUMC | 37 | LDT PCR | Enterovirus | 33.0 | Enterovirus | 33.9 | **YES** |
| LUMC | 38 | LDT PCR | Enterovirus | 30.6 | Enterovirus | 32.5 | **YES** |
| LUMC | 39 | LDT PCR | Enterovirus | 31.8 | Enterovirus | 35.4 | **YES** |
| LUMC | 40 | LDT PCR | Enterovirus | 28.7 | Enterovirus | 31.5 | **YES** |
| AUMC | 41 | LDT PCR | *H. influenza* | 36.3 | *H. influenza* | 32.9 | **YES** |
| AUMC | 42 | LDT PCR | *H. influenza* | 17.2 | *H. influenza* | 14.2 | **YES** |
| AUMC | 43 | LDT PCR | *H. influenza* | 26.8 | *H. influenza* | 27.3 | **YES** |
| AUMC | 44 | LDT PCR | *H. influenza* | 37.7 | Cartridge failure | NA | **EXCLUDED** |
| AUMC | 45 | LDT PCR | *H. influenza* | 32.0 | *H. influenza* | 29.6 | **YES** |
| LUMC | 46 | Culture | *H. influenza* | NA | *H. influenza* | 18.4 | **YES** |
| LUMC | 47 | Culture | *H. influenza* | NA | *H. influenza* | 14.6 | **YES** |
| LUMC | 48 | Culture | *H. influenza* | NA | *H. influenza* | 17.6 | **YES** |
| LUMC | 49 | LDT PCR | HHV-6 | 36.1 | HHV-6 | 31.1 | **YES** |
| LUMC | 50 | LDT PCR | HHV-6 | 31.5 | HHV-6 | 33.9 | **YES** |
| LUMC | 51 | LDT PCR | HHV-6 | 31.7 | HHV-6 | 31.1 | **YES** |
| LUMC | 52 | LDT PCR | HSV-1 | 28.6 | HSV-1 | 27.2 | **YES** |
| LUMC | 53 | LDT PCR | HSV-1 | 29.1 | HSV-1 | 27.5 | **YES** |
| LUMC | 54 | LDT PCR | HSV-1 | 31.4 | HSV-1 | 33.5 | **YES** |
| LUMC | 55 | LDT PCR | HSV-1 | 25.6 | HSV-1 | 25.4 | **YES** |
| LUMC | 56 | LDT PCR | HSV-1 | 33.4 | HSV-1 | 32.6 | **YES** |
| LUMC | 57 | LDT PCR | HSV-1 | 28.7 | HSV-1 | 28.2 | **YES** |
| LUMC | 58 | LDT PCR | HSV-2 | 36.6 | HSV-2 | 35.0 | **YES** |
| LUMC | 59 | LDT PCR | HSV-2 | 28.6 | HSV-2 | 27.0 | **YES** |
| LUMC | 60 | LDT PCR | HSV-2 | 32.8 | HSV-2 | 30.6 | **YES** |
| LUMC | 61 | LDT PCR | HSV-2 | 35.2 | HSV-2 | 33.8 | **YES** |
| AUMC | 62 | Culture | *L. monocytogenes* | NA | *L. monocytogenes* | 26.5 | **YES** |
| AUMC | 63 | Culture | *L. monocytogenes* | NA | *L. monocytogenes* | 28.5 | **YES** |
| LUMC | 64 | LDT PCR | *L. monocytogenes* | 30.2 | *L. monocytogenes* | 33.1 | **YES** |
| LUMC | 65 | LDT PCR | *L. monocytogenes* | 32.8 | *L. monocytogenes* | 32.5 | **YES** |
| LUMC | 66 | LDT PCR | *L. monocytogenes* | 17.5 | *L. monocytogenes* | 16.6 | **YES** |
| LUMC | 67 | LDT PCR | *L. monocytogenes* | 28.7 | *L. monocytogenes* | 31.0 | **YES** |
| LUMC | 68 | LDT PCR | *L. monocytogenes* | 35.8 | Negative | NA | **NO** |
| AUMC | 69 | LDT PCR | *N. meningitidis* | 30.1 | *N. meningitidis* | 26.6 | **YES** |
| AUMC | 70 | LDT PCR | *N. meningitidis* | 25.0 | *N. meningitidis* | 23.5 | **YES** |
| AUMC | 71 | LDT PCR | *N. meningitidis* | 27.0 | *N. meningitidis* | 24.6 | **YES** |
| AUMC | 72 | LDT PCR | *N. meningitidis* | 27.0 | *N. meningitidis* | 26.6 | **YES** |
| AUMC | 73 | LDT PCR | *N. meningitidis* | 21.7 | *N. meningitidis* | 22.9 | **YES** |
| LUMC | 74 | LDT PCR | *N. meningitidis* | 17.9 | *N. meningitidis* | 17.3 | **YES** |
| LUMC | 75 | LDT PCR | *N. meningitidis* | 17.6 | *N. meningitidis* | 17.3 | **YES** |
| LUMC | 76 | LDT PCR | *N. meningitidis* | 18.2 | *N. meningitidis* | 17.5 | **YES** |
| LUMC | 77 | LDT PCR | *N. meningitidis* | 26.1 | *N. meningitidis* | 26.7 | **YES** |
| LUMC | 78 | LDT PCR | *N. meningitidis* | 27.2 | *N. meningitidis* | 26.4 | **YES** |
| LUMC | 79 | LDT PCR | Parechovirus | 37.1 | Parechovirus | 36.0 | **YES** |
| LUMC | 80 | LDT PCR | Parechovirus | 21.2 | Parechovirus | 31.1 | **YES** |
| LUMC | 81 | LDT PCR | Parechovirus | 31.5 | Parechovirus | 33.6 | **YES** |
| LUMC | 82 | LDT PCR | Parechovirus | 30.1 | Parechovirus | 32.0 | **YES** |
| LUMC | 83 | LDT PCR | Parechovirus | 26.8 | Parechovirus | 30.5 | **YES** |
| AUMC | 84 | IS-pro | *S. agalactiae* | NA | *S. agalactiae* | 34.9 | **YES** |
| AUMC | 85 | LDT PCR | *S. pneumoniae* | 23.0 | *S. pneumoniae* | 17.5 | **YES** |
| AUMC | 86 | LDT PCR | *S. pneumoniae* | 40.0 | Negative | NA | **NO** |
| AUMC | 87 | LDT PCR | *S. pneumoniae* | 25.0 | Cartridge failure | NA | **EXCLUDED** |
| AUMC | 88 | LDT PCR | *S. pneumoniae* | 31.0 | *S. pneumoniae* | 28.1 | **YES** |
| AUMC | 89 | LDT PCR | *S. pneumoniae* | 18.0 | *S. pneumoniae* | 15.4 | **YES** |
| LUMC | 90 | LDT PCR | *S. pneumoniae* | 26.7 | *S. pneumoniae* | 26.4 | **YES** |
| LUMC | 91 | LDT PCR | *S. pneumoniae* | 23.9 | *S. pneumoniae* | 21.7 | **YES** |
| LUMC | 92 | LDT PCR | *S. pneumoniae* | 13.9 | *S. pneumoniae* | 14.4 | **YES** |
| LUMC | 93 | LDT PCR | *S. pneumoniae* | 26.0 | *S. pneumoniae* | 23.8 | **YES** |
| LUMC | 94 | LDT PCR | *S. pneumoniae* | 14.1 | *S. pneumoniae* | 15.2 | **YES** |
| LUMC | 95 | LDT PCR | *S. pneumoniae* | 37.8 | *S. pneumoniae* | 37.7 | **YES** |
| LUMC | 96 | LDT PCR | *S. pneumoniae* | 33.7 | *S. pneumoniae* | 34.2 | **YES** |
| AUMC | 97 | Culture | *S. pyogenes* | NA | *S. pyogenes* | 19.7 | **YES** |
| AUMC | 98 | Culture | *S. pyogenes* | NA | *S. pyogenes* | 21.8 | **YES** |
| LUMC | 99 | Culture | *S. pyogenes* | NA | *S. pyogenes*, VZV (CT 35.9) | 20.9 | **NO** |
| LUMC | 100 | LDT PCR | VZV | 36.1 | VZV | 34.9 | **YES** |
| LUMC | 101 | LDT PCR | VZV | 29.3 | VZV | 30.0 | **YES** |
| LUMC | 102 | LDT PCR | VZV | 32.6 | VZV | 33.6 | **YES** |
| LUMC | 103 | LDT PCR | VZV | 29.8 | VZV | 30.7 | **YES** |
| LUMC | 104 | LDT PCR | VZV | 34.0 | VZV | 34.5 | **YES** |
| LUMC | 105 | LDT PCR | VZV | 29.8 | VZV | 29.8 | **YES** |
| LUMC | 106 | LDT PCR | VZV | 33.0 | VZV | 33.2 | **YES** |
| LUMC | 107 | LDT PCR | VZV | 30.1 | VZV | 29.5 | **YES** |
| LUMC | 108 | LDT PCR | VZV | 32.7 | VZV | 34.8 | **YES** |
| LUMC | 109 | LDT PCR | VZV | 30.3 | VZV | 31.7 | **YES** |
| LUMC | 110 | LDT PCR | VZV | 28.6 | VZV | 28.1 | **YES** |
| AUMC | Specificity_1 | IS-pro | Negative | NA | Negative | NA | **YES** |
| AUMC | Specificity_2 | IS-pro | *S. bovis* group | NA | Negative | NA | **YES** |
| AUMC | Specificity_3 | IS-pro | *S. bovis* group | NA | Negative | NA | **YES** |
| AUMC | Specificity_4 | IS-pro | *S. dysgalactiae* | NA | Negative | NA | **YES** |

AUMC – Amsterdam University Medical Center, LUMC – Leiden University Medical Center.

**Table S2: Differences in C_T_ values between the LDT and QS in enterovirus and parechovirus positive CSF samples.**

| **Sample ID** | **Target** | **QS-ME result** | **LDT result** | **ΔC_T_** | **Genotype** |
| --- | --- | --- | --- | --- | --- |
| 17 | Enterovirus | 31.9 | 29.9 | -2.0 | Not performed |
| 18 | Enterovirus | 34.3 | 31.0 | -3.3 | Echovirus E30 |
| 19 | Enterovirus | 33.4 | 31.7 | -1.7 | Not performed |
| 20 | Enterovirus | 37.2 | 28.4 | -8.8 | Echovirus E30 |
| 21 | Enterovirus | 39.4 | 29.2 | -10.2 | Echovirus E30 |
| 22 | Enterovirus | 33.6 | 31.6 | -2.0 | Echovirus E6 |
| 23 | Enterovirus | 36.4 | 27.3 | -9.1 | Echovirus E30 |
| 24 | Enterovirus | 35.8 | 33.9 | -1.9 | Echovirus E6 |
| 25 | Enterovirus | 34.9 | 32.6 | -2.3 | Not performed |
| 26 | Enterovirus | 38.2 | 34.8 | -3.4 | Subtyping failed |
| 27 | Enterovirus | 33.7 | 32.4 | -1.3 | Not performed |
| 28 | Enterovirus | 37.9 | 29.8 | -8.1 | Echovirus E30 |
| 29 | Enterovirus | 34.9 | 33.0 | -1.9 | Not performed |
| 30 | Enterovirus | 34.2 | 32.2 | -2.0 | Not performed |
| 31 | Enterovirus | 37.0 | 35.6 | -1.4 | Echovirus E30 |
| 32 | Enterovirus | 35.8 | 32.4 | -3.4 | Echovirus E30 |
| 33 | Enterovirus | 23.3 | 23.2 | -0.1 | Coxsackievirus B5 |
| 34 | Enterovirus | 26.7 | 25.6 | -1.1 | Echovirus E1 |
| 35 | Enterovirus | 26.6 | 24.7 | -1.9 | Echovirus E9 |
| 36 | Enterovirus | 31.5 | 30.5 | -1.0 | Not performed |
| 37 | Enterovirus | 33.9 | 33.0 | -0.9 | Echovirus E30 |
| 38 | Enterovirus | 32.5 | 30.6 | -1.9 | Echovirus E13 |
| 39 | Enterovirus | 35.4 | 31.8 | -3.6 | Not performed |
| 40 | Enterovirus | 31.5 | 28.7 | -2.8 | Not performed |
| 79 | Parechovirus | 36.0 | 37.1 | 1.1 | Not performed |
| 80 | Parechovirus | 31.1 | 21.2 | -9.9 | Not performed |
| 81 | Parechovirus | 33.6 | 31.5 | -2.1 | Not performed |
| 82 | Parechovirus | 32.0 | 30.1 | -1.9 | Not performed |
| 83 | Parechovirus | 30.5 | 26.8 | -3.7 | Not performed |

Rows in grey represent samples with more than three C_T_ value differences obtained from the QS-ME versus LDT. Where a genotype is depicted in white, this was determined as part of the routine diagnostic workflow, whereas in grey additional genotyping experiments were performed to investigate a potential relationship between subtypes and C_T_ value differences.
